# Editorial: Interaction between affect and memory in the brain: From basic mechanisms to clinical implications

**DOI:** 10.3389/fnbeh.2023.1120282

**Published:** 2023-01-24

**Authors:** Yuta Katsumi, Lycia D. de Voogd, Carlos Ventura-Bort, Wei Liu, Shaozheng Qin

**Affiliations:** ^1^Frontotemporal Disorders Unit, Department of Neurology, Massachusetts General Hospital and Harvard Medical School, Boston, MA, United States; ^2^Center for Cognitive Neuroimaging, Donders Institute for Brain Cognition and Behaviour, Radboud University, Nijmegen, Netherlands; ^3^Behavioral Science Institute, Radboud University, Nijmegen, Netherlands; ^4^Department of Biological Psychology and Affective Science, Faculty of Human Sciences, University of Potsdam, Potsdam, Germany; ^5^Key Laboratory of Adolescent Cyberpsychology and Behavior (CCNU), Ministry of Education, Wuhan, China; ^6^Key Laboratory of Human Development and Mental Health of Hubei Province, School of Psychology, Central China Normal University, Wuhan, China; ^7^State Key Laboratory of Cognitive Neuroscience and Learning & IDG/McGovern Institute for Brain Research, Beijing Normal University, Beijing, China; ^8^Chinese Institute for Brain Research, Beijing, China

**Keywords:** emotion, episodic memory, encoding, consolidation, retrieval, functional networks

Stressful and arousing experiences produce lasting memories. Such memory enhancement is supported by distinct mechanisms over different timescales, from immediate effects of arousal modulating attentional, sensory, and mnemonic processes during encoding (Bradley et al., 1992; Cahill and McGaugh, 1995; Buchanan et al., 2006) to preferential consolidation of arousing information over time (McGaugh, 2018). Previous animal and human studies consistently demonstrated that these mechanisms depend in large part on the amygdala, hippocampus, and their interaction (Strange et al., 2014; Bocchio et al., 2017; McGaugh, 2018; Costa et al., 2022). In addition, selective processing of information under arousal also involves widespread neocortical and subcortical regions (Mather et al., 2016). These include regions part of the salience network (Seeley et al., 2007), a domain-general functional network that is thought to be critical for the prioritization and integration of incoming sensory signals (Sepulcre et al., 2012; Katsumi et al., 2022). As such, arousal-related modulation of memory can also manifest at the whole-brain level (see also Katsumi and Moore, this issue), presumably due to diffuse influences exerted by the locus coeruleus-norepinephrine system across the brain (Mather et al., 2016). Selective encoding and consolidation of memories for arousing events also have profound clinical implications. For instance, post-traumatic stress disorder (PTSD) is typically characterized by involuntary and intrusive recollection of unwanted memories (Brewin et al., 1996; Brewin, 2007). These symptoms following traumatic experiences have been linked to dysfunctional neural mechanisms subserving the interaction between affect and memory, including the involvement of the amygdala, hippocampus, and prefrontal cortical regions, among others (Pitman et al., 2012; Dolcos et al., 2020). However, recent evidence suggests that there exists considerable heterogeneity in the diagnosis of PTSD, which may explain limited benefits of available psychotherapies and pharmacotherapies for this disorder despite extensive research (Neria, 2021). Clarification of the effects of affective arousal on episodic memory processes would therefore be essential in advancing our understanding of the development of such disorders and to potentially pave the way for refined intervention and treatment. This Research Topic features a collection of papers^1^ addressing timely questions related to the interplay between affective and mnemonic processes using a variety of methodological approaches, experimental contexts, and subject populations.

Two papers investigated the effects of experimental manipulations that modulate the impact of affective information on episodic memory. Bouvarel et al. tested young adults to examine how the focality of emotional content depicted in pictures affects their recognition memory. The authors report that pictures containing a central emotional component in the context of peripheral neutral features (“focal” emotional pictures) were less remembered than those containing neutral information only, when the peripheral neutral features were used as retrieval cues and the visual complexity of pictures was high (Study 3). Pictures containing emotional components throughout (“diffuse” emotional pictures) were less remembered than neutral pictures, suggesting that emotion may impair memory depending on the experimental context. Tamera et al. manipulated young and older adults' moods using video clips to investigate how their induced moods affect performance on subsequent memory recall. The authors report that young adults showed a persistent negativity bias (i.e., better memory for negative pictures) irrespective of mood conditions, whereas older adults did not and instead showed a slight positivity preference. The authors interpret the pattern of older adults' memory performance as being consistent with mood incongruence effects and an age-related positivity effect.

Two other papers investigated the mechanisms associated with memory inhibition using event-related potentials. Kissler and Hauswald asked participants to view a series of pictures showing an angry or neutral face, each of which was followed by a cue to either remember or forget the stimulus; participants' memory for faces was later tested in a recognition task. The authors report that recognition accuracy was higher for to-be-remembered than to-be-forgotten items. Intentionally remembering faces elicited larger late centro-parietal positivity than forgetting them. Intentional forgetting also produced larger late frontal positivity than remembering, uniquely for angry faces. The authors suggest that forgetting angry faces may be more cognitively demanding. Bublatzky et al. asked participants to learn reward contingencies by choosing between two behavioral options per trial, which were reversed after reaching a threshold. This task was performed in either a threat or safety context, where participants received aversive shocks in the former. While the effect of learning context was minimal, the authors report reduced feedback-related fronto-central P3 amplitudes during a threat compared with safety context. The authors interpret this effect as reflecting the interference effects of contextual threat on attentional and memory processes.

Two additional papers examined the mechanisms underlying the effect of affective information on episodic memory using functional magnetic resonance imaging (fMRI). Bradley and Sambuco reviewed fMRI evidence and discuss several issues related to the involvement of the amygdala in emotional memory. The authors argue that, while processing of emotional (vs. neutral) pictures is consistently associated with robust amygdala activation, retrieval of emotional (vs. neutral) autobiographical memories is not. This suggests that the amygdala may be involved in encoding but not retrieval of emotional memories. The authors also discuss issues related to inconsistent definitions of fMRI contrasts in studies examining emotional memory retrieval, some of which may reflect differences in emotionality rather than memory. Katsumi and Moore analyzed resting-state fMRI data collected from participants across the adult lifespan to identify aspects of intrinsic functional connectivity that are associated with the affective enhancement of episodic memory. The authors report that the affective enhancement of memory was associated with connectivity patterns of several large-scale cortical functional networks and a few subcortical structures. The authors conclude that the affective enhancement of memory may be characterized as a whole brain phenomenon, consistent with other views of arousal effects on cognition (e.g., Mather et al., 2016).

Lastly, two papers examined the mechanisms underlying dysfunctional affective and memory-related processes in patients with PTSD. Marlatte et al. examined the relationship between performance on hippocampal-dependent tasks and brain structural integrity. Using a multiple factor analysis, the authors report two components that summarized the data, which were characterized by impaired spatial processing in patients coupled with abnormal gray and white matter integrity within a network of brain regions including the hippocampus. Thome et al. analyzed resting-state fMRI and pulse data collected from patients with PTSD and its dissociative subtype (PTSD + DS). The authors investigated the relationship between intrinsic functional connectivity of the pedunculopontine nuclei (PPN; a central node of the reticular activation system) and heart rate variability. The authors report that, whereas patients with PTSD exhibited a positive relationship between PPN-amygdala connectivity and heart rate variability, those with PTSD + DS showed a negative relationship. The authors interpret this effect as potentially reflecting hyper- and more blunted arousal states in PTSD and PTSD + DS, respectively.

Collectively, these reports provide insights into the mechanisms underlying the complex interaction of affective and memory-related processes in healthy and clinical populations. Such diverse contributions enable an integrative understanding of the mechanisms associated with affect-memory interactions observed in broad contexts. We believe that the present findings represent an important step toward achieving the goal of developing biomarkers that can be targeted by therapeutic interventions designed to modulate the dynamics of affect-memory interactions when desirable. Building upon this Research Topic, future work should address issues pertinent to this major goal, with a focus on better understanding the heterogeneity and reliability of the mechanisms underlying affect-memory interactions. Recent research shows that test-retest reliability of the enhancement of memory by affective arousal is rather low—i.e., confidence intervals of test-retest correlations across participants over 7–10 weeks falling consistently below *r* = 0.5 (Schümann et al., 2020). This suggests that the magnitude of affective enhancement of memory may be in part state-dependent and influenced by factors like mood and anxiety (see also Tamera et al., this issue). Much of current knowledge about the neural mechanisms of the affective enhancement of memory is based on studies utilizing various neuroimaging techniques including task-related fMRI. Yet, the test-retest reliability of brain activation identified in fMRI studies using common experimental tasks, including emotion processing tasks, has been shown to be poor (as low as intraclass correlation = 0.067; Elliott et al., 2020). Altogether, these findings point to the need for clarifying robust biomarkers that may be targeted by interventions to promote adaptive memory processes depending on the context. One promising approach may be to measure and model variation *within* each participant over repeated measures (e.g., trial-level fluctuations) in brain activity; this would enable isolation of stable signal variance specific to each individual participant (Elliott et al., 2021; Westlin et al., in press), which may inform the design of brain stimulation or pharmacological interventions with maximal efficacy.

## Author contributions

YK wrote the initial draft of the manuscript. All authors contributed to the conception of the Research Topic, edited, revised, and approved the final version of the manuscript.

## Footnotes

^1^In this editorial, we describe each contribution using the original authors' language as much as possible. This does not necessarily mean that we agree with the way certain psychological constructs are operationalized and discussed.
